# An international survey of perceptions of the 2014 FIFA World Cup: National levels of corruption as a context for perceptions of institutional corruption

**DOI:** 10.1371/journal.pone.0222492

**Published:** 2019-09-27

**Authors:** John B. Nezlek, David B. Newman, Astrid Schütz, Roy F. Baumeister, Joanna Schug, Mohsen Joshanloo, Paulo N. Lopes, Nicholas P. Alt, Marzena Cypryańska, Marco Depietri, Oleg Gorbaniuk, Pascal Huguet, Konstantinos Kafetsios, Selda Koydemir, Peter Kuppens, Sanghee Park, Alvaro San Martin, Juliette Schaafsma, Dora Simunovic, Kunihiro Yokota

**Affiliations:** 1 Institute of Psychology, SWPS University of Social Sciences and Humanities, Poznań, Poland; 2 Department of Psychological Sciences, College of William and Mary, Williamsburg, Virginia, United States of America; 3 Department of Psychology, University of Southern California, Los Angeles, California, United States of America; 4 Department of Psychology, University of Bamberg, Bamberg, Germany; 5 School of Psychology, University of Queensland, Brisbane, Queensland, Australia; 6 Department of Psychology, Keimyung University, Daegu, South Korea; 7 Católica Lisbon School of Business and Economics, Catholic University of Portugal, Lisbon, Portugal; 8 Department of Psychology, University of California, Los Angeles, California, United States of America; 9 Department of Psychology, SWPS University of Social Sciences and Humanities, Warsaw, Poland; 10 Language Centre, University of Bamberg, Bamberg, Germany; 11 Institute of Psychology, John Paul II Catholic University of Lublin, Lublin, Poland; 12 National Centre for Scientific Research, Blaise Pascal University, Clermont-Ferrand, France; 13 Department of Psychology, University of Crete. Rethymno, Greece; 14 Research unit for Quantitative Psychology and Individual Differences, KU Leuven, Leuven, Belgium; 15 Department of Psychology, Chungbuk National University, Cheongju-si, South Korea; 16 Department of Managing People in Organizations, University of Navarra, Pamplona, Spain; 17 Department of Communication and Cognition, Tilburg University, Tilburg, Netherlands; 18 Bremen International Graduate School of Social Sciences, Jacobs University, Bremen, Germany; 19 Department of Evolutionary Studies of Biosystems, The Graduate University for Advanced Studies, Miura, Japan; Mälardalen University, SWEDEN

## Abstract

We conducted a survey about the 2014 FIFA World Cup that measured attitudes about FIFA, players, and officials in 18 languages with 4600 respondents from 29 countries. Sixty percent of respondents perceived FIFA officials as being dishonest, and people from countries with less institutional corruption and stronger rule of law perceived FIFA officials as being more corrupt and less competent running the tournament than people from countries with more corruption and weaker rule of law. In contrast, respondents evaluated players as skilled and honest and match officials as competent and honest. We discuss the implications of our findings for perceptions of corruption in general.

## Introduction

Corruption is a worldwide problem. It not only siphons resources away from those who earn and deserve them, but it can also distort economic incentives so as to impair growth. It weakens public trust in institutions, thereby damaging the view that authority is exercised legitimately. Corruption can also damage the social fabric, so that instead of a productive, cooperative society, people live in cynical, fragmented, uncooperative collections of individuals. See Dimant and Tosato [1] for a review of the causes and consequences of corruption.

Corruption has been studied primarily in terms of governmental corruption (e.g., World Bank, Kaufman, Kraay, & Mastruzzi, [2]), but other institutions can also be corrupt. Perceptions of corruption in government can be confounded with patriotism and political partisanship, so it can be instructive to examine perceived corruption in a non-governmental organization. The present investigation studied people’s perceptions of corruption in FIFA (the Fédération Internationale de Football Association). FIFA is a huge international organization that oversees one of the world’s most celebrated and widely followed sports events, the World Cup tournament of football. Football is referred to as soccer in some places, but given that football is the term used by most people internationally and is the term that is part of FIFA, we will refer to the sport as football. National teams from the 208 member countries of FIFA compete for two years, after which the 32 teams with the best records meet for a tournament to determine the world champion. The worldwide interest in this tournament made it possible to compare perceptions across many different countries.

We surveyed citizens in multiple countries about their perceptions of corruption in connection with the 2014 World Cup, including how they perceived the players, the referees, and FIFA itself. Of particular interest was the question of how general (governmental) corruption in a country was related to how the residents of that country perceived corruption in football. Opposing hypotheses, corresponding roughly to assimilation and contrast, were entertained. We should note that the present study concerns perceptions of the men’s World Cup. FIFA also organizes a World Cup for women, but to our knowledge, there have not been allegations of corruption in FIFA concerning the women’s World Cup.

### Is football corrupt?

The question of corruption in FIFA may strike informed readers as quaint, given that the last few years have seen highly publicized trials and dismissals of top officials; however, the present survey preceded much of this negative publicity. Specifically, the data were collected in 2014, during and shortly after the tournament’s final rounds. The tournament was conducted in Brazil, whose government corruption scandals have also been prominent in the news—but again this was just starting in 2014.

Still, the international football news had already contained some signs that FIFA had a corruption problem. A decade earlier, Sugden and Tomlinson [3] wrote a widely acclaimed exposé, “Badfellas” that described systematic corruption in world football, with a focus on FIFA. The book was so critical of FIFA that FIFA tried to ban its publication [4].

To be sure, worse was to come, and it became reasonable to assume that FIFA was indeed corrupt. In 2014 a special investigator, Michael Garcia, a former US federal prosecutor, was appointed to investigate the awarding of the 2018 and 2022 World Cups to Russia and Qatar. Garcia resigned after FIFA decided not to release the report [5]. Moreover, numerous FIFA officials have been charged by the US and Swiss governments with corruption [6]. Following the 2014 Cup (the focus of this study) Sugden and Tomlinson [4] updated their 2003 book to include a discussion of the removal of Sepp Blatter, the president of FIFA, on suspicion of corruption.

Moreover, charges of corruption have not been limited to the international governing body (FIFA). For example, what is often called the Simmons Report [7] concluded explicitly and directly that both Jack Warner (a former president of the Confederation of North, Central American and Caribbean Association) and Chuck Blazer (a former General Secretary of CONCACAF) had committed “fraud against CONCACAF” (p. vii). Blazer died in July of 2017 after cooperating with the US FBI, and Warner is currently (January, 2019) fighting extradition to the US to stand trial on corruption charges [8].

A particular issue is whether perceptions of corruption would include players and referees. Referees are expected to be impartial, but in many sports the fairness and impartiality of officials have been questioned. In a classic study of motivated social perception, Hastorf and Cantril [9], students counted rule infractions in a taped (American) college football game, and these tallies were systematically biased in favor of their own college’s team. Given such biases among fans, even a perfectly fair and impartial official can be suspected of bias by some viewers.

As to the players themselves, there are occasional accusations of players being paid to lose games, though whether that occurs at the high level of competition that exists in the World Cup is not clear. Still, some unfair play has been subject to commentary, most notably when players react to minor physical contact by falling down dramatically (often informally called “flopping” of “diving”) in the hope that the official will charge the other team with a penalty. Based on Hastorf and Cantril [9], it seemed reasonable to predict that participants would rate corruption higher on their most disliked team than on their favorite team and would suspect referees of being biased against their favorite team.

### Competing hypotheses

Our primary focus was on how perceptions of corruption in football would be related to corruption closer to home. Such relationships can be understood in terms of comparisons between perceptions of corruption in football, defined as a target in research on comparisons processes, and the corruption in one’s home country, defined as a standard in research on comparison processes. Broadly speaking, comparisons between a target and a standard can reflect either assimilation or contrast effects. Assimilation or “carry-over” effects tend to occur when accessible information is used to form a mental representation of a target of judgment, whereas contrast or “backfire” effects occur when accessible information is used to form a mental representation of the standard against which the target is being compared [10]. In terms of comparisons per se, assimilation results in perceiving the target and the standard to be similar, whereas contrast results in perceiving the target and the standard to be different.

In general terms, judgment may be influenced by both chronically and temporarily accessible information [11, 12], and such information can have assimilation or contrast effects, depending on the context [10]. For example, people’s mood (a type of temporarily accessible information) can influence people’s judgments about their satisfaction with life [13], e.g., the more positive a person’s immediate mood is the more positively they view their life overall. This is an assimilation effect. On the other hand, if a standard becomes more salient, a contrast effect occurs and targets and standards are seen to differ [14]. For example, watching attractive actors in a movie can lower the perceived attraction of one’s romantic partner [15]. In this example, the standard (actors in a movie) are salient, and this leads people to see their partners (the targets) as dissimilar (less attractive).

In the present study, it was possible to predict either a positive or negative relationship between a country’s level of corruption and perceptions of corruption in FIFA. Importantly, we did not rely on citizens’ own ratings of corruption in their country, partly because we were concerned that answering questions about corruption in FIFA might alter ratings of corruption of one’s own country, or vice versa. That is, we were concerned about any effects of temporarily accessible information. Hence we relied on independent ratings of country-by-country corruption, which are regularly supplied and updated by the World Bank [2]. Thus, our study examined relationships between societal corruption, as assessed by the World Bank, and subjective perceptions of corruption in football, as rated by our participants.

The assimilation hypothesis is that citizens in highly corrupt countries would be most likely to perceive and condemn FIFA as corrupt. Multiple cognitive processes could contribute to this effect. People who live in corrupt countries are presumably familiar with corruption and can recognize its telltale signs more readily than those who do not encounter corruption very much. They may expect and assume that corruption is widespread and perhaps that it may be a simple fact of life. In this case, frequent exposure to corruption becomes chronically accessible, and this information could be used when evaluating the target, namely FIFA.

A contrast effect leads to the opposite relationship. People who live in corrupt societies may gradually cease to notice it or become desensitized to it, whereas in non-corrupt countries, people may expect fairness and transparency, and so indications of corruption will stand out as serious violations of expectancies. This information would be used to form the standard, to which the target would be compared.

Anecdotally, one of us has heard several highly educated friends firmly assert that today’s United States is a highly corrupt society—yet when asked whether they themselves had ever paid a bribe, they each said no. The World Bank rates the United States low in corruption, so these perceptions of corruption suggest a contrast effect. Then again, people who have never paid bribes may be less prone to notice signs of corruption (including subtle requests for bribes) than others.

Thus, it was possible to predict either a positive or negative relationship between a country’s level of corruption and how much its football fans perceive corruption in FIFA. Hypotheses about perceived corruption in referees and players were more tentative. The assimilation hypothesis assumes that corruption is widespread, and so it might well extend to seeing referees and players as corrupt—though news reports had not begun to implicate referees or players, unlike FIFA itself. The lack of such reports would especially undermine the contrast process, which relies on some evidence of corruption to stimulate the outraged reaction. Last, bias in perception (as shown by Hastorf and Cantril [9]) would incline people to rate their most disliked team as more corrupt than their favorite team.

### The present study

Given the international nature of the World Cup, we aimed to obtain an international sample. We collected data anonymously via a website that was available in 18 languages. The survey was brief and took less than 5 minutes to complete, something we believed enhanced cooperation. Participants answered background questions about themselves, (demographic questions and satisfaction with life) and questions about the World Cup (the players, officials, the teams, and FIFA). These data provided a basis to examine relationships between perceptions of the World Cup and a country level measure of corruption that we created using widely accepted indices made available by the World Bank.

We asked questions about the players, officials, and teams to control for the possibility that perceptions of corruption in FIFA reflected some type of broader negative evaluation of football or dissatisfaction with life in general. Although our primary focus was on perceptions of corruption in FIFA, these additional measures allowed us to determine if relationships between country-level corruption and perceptions of corruption in FIFA would vary after controlling for perceptions of different aspects of football. If the relationship between country-level corruption and perceptions of corruption in FIFA did not change after controlling for these other measures this would suggest that perceptions of corruption in FIFA reflected evaluations of FIFA per se and not a more generalized perception of corruption or more generalized positive or negative evaluations. This can be thought of as examining the divergent validity of our measure of perceived corruption in FIFA.

## Method

### Respondents and sampling procedure

Our goal was to obtain as broad a sample as possible from as many countries as possible. Respondents were made aware of the study in various ways such as newspaper articles, press releases, postings on blogs related to football, requests to football associations, and so forth. We focused on countries that had a team in the Cup, but we did not limit respondents to those countries. We relied in part on a “snowball” approach wherein those who took the survey would recommend or encourage others to do the same. The difficulties we had in obtaining respondents and the limitations imposed by our sample are discussed later.

The finals of the 2014 FIFA World Cup began on 12 June 2014 and ended on 13 July 2014. The group stages ended on 26 June, and the knock-out rounds began on 28 June. The survey was first posted on 10 July (just before the semi-finals), and we stopped collecting data on 11 August, although it should be noted that 92% of our final sample had responded by 20 July. Setting the beginning and end dates of the data collection window reflected two considerations. First, we wanted the bulk of the tournament to have been completed to maximize the information on which respondents could base their responses. Second, we wanted to collect data before the tournament had ended and while it was still in the public conversation (media attention, etc.), assuming that this would result in a larger number of respondents. In terms of the end date, we felt that a few weeks was the maximum time that people’s memories would remain reasonably fresh. Moreover, there were few new responses each day after 1 August.

The study protocol was approved by the College of William Mary Committee for the Protection of Human Subjects, protocol: PHSC-2014-07-03-9689-jbnezl. Participants provided written consent by indicating this on the first page of the survey.

### Survey instrument

The survey instrument was developed by the research team with specific attention to wording items in ways that would provide the easiest translations from English (the base language) to other languages. The construction of the items and responses was also guided by the recommendations offered by Gehlbach and Brinkworth [16]. The resulting English base version was translated and back-translated by individuals who were fluent in English and another language. Participants were able to complete the survey in one of the following languages: English, Portuguese (Brazilian), Dutch, French, German, Greek, Italian, Korean, Persian, Polish, Portuguese (Portugal), Russian, Spanish, Turkish, Ukrainian, Japanese, Croatian, and Czech.

People provided their responses via a secure website with a greeting and introduction in their language of choice. The survey had six parts: background questions about the respondent and measures of respondents’ perceptions of the players, the officials, of their favorite and least favorite teams, and of FIFA. A copy of the English base version of the survey is available as part of the Supplemental Materials (https://osf.io/z5rbp/?view_only=2b25601521fd45db865964c45af86553), and the items that are discussed in this paper are provided below. Unless indicated otherwise, all responses were made on 5-point scales in which 5 represented more, greater, stronger, and so forth, and 1 represented less, fewer, or weaker. Given the lack of research about this topic, although questions were organized in terms of topics, we did not treat questions about the same topic as measured variables of latent constructs. Our analyses and consequent discussion focuses on individual measures.

We should note that given the lack of quantitative research on perceptions of corruption in sport we included measures of numerous constructs that might call into question the validity of our analyses about relationships between perceptions of corruption in FIFA and country-level corruption. We could not be certain what types of perceptions would be related to perceptions of corruption in FIFA in ways that would undermine the strength of the inference of the analyses regarding our primary hypothesis of interest. For example, would people’s evaluations of the players or the officials be related to perceptions of FIFA, and if so, what aspects of these evaluations would be related, competence, motivation, or honesty? Although we had no compelling reasons to expect that controlling for individual differences in these measures would compromise our results, we included them to address such possibilities.

These additional measures also addressed issues of divergent validity. For example, if the relationship between country-level corruption and perceptions of corruption in FIFA did not change after controlling for individual differences in perceptions of corruption among referees, this would suggest that perceptions of corruption among FIFA is a construct that is distinct from perceptions of corruption among referees. The data we collected also allowed us to address the issue that perceptions of corruption in FIFA would primarily reflect a sort of global good-bad judgment rather than a judgment about FIFA per se. We included good-bad evaluations of various components of the Cup to do this.

Respondents began by answering two background questions: How interested were you in the World Cup, and overall, how satisfied are you with your life in general. We asked the question about interest in the Cup to examine how engaged participants were with the Cup (did they care about the competition) and to control for the possibility that perceptions of corruption of FIFA reflected how much people cared about the Cup, e.g., “I don’t care about the Cup, so why think about corruption in FIFA?” The question about life satisfaction was included to control for the possibility that evaluations of FIFA reflected how satisfied participants were with their lives (i.e., evaluations of their lives) rather than evaluations of FIFA per se.

They then answered three questions about the players, each of which started with the stem “Thinking of all the players on all the teams …” The questions asked: how much do you think the players cared about winning, how skilled do you think the players were, and how much of a problem do you think “flopping,” "diving," and other ways of feigning a foul were. These questions were included to examine the possibility that participants’ evaluations of FIFA reflected their overall evaluations of the players. These questions concerned players’ motivation, their skills, and one of the most obvious indicators of players’ bending (if not breaking) the rules of the game.

Respondents also answered a series of questions about their most favorite and about their least favorite team. For present purposes, we analyzed two of these questions: “Do you think any of the players on this team were corrupt?” and “In general, how neutral do you think the officials were in the matches involving this team?” Responses to the second question were made on a 5-point scale with endpoints labelled 1 = were strongly against this team 5 = were strongly in favor of this team, and a midpoint labelled 3 = were neutral. These items were included to examine the possibility that perceptions of corruption in FIFA were colored by respondents’ perceptions of corruption among players in two salient teams (favored and least favored) or perceptions of possible corruption among officials who refereed matches involving these teams.

Then there were five questions about the referees: Overall, please rate the quality of the referees, How accurate were the referees in terms of calling offsides, In general, what do you think of how the referees called fouls, In general, how well do you think the referees noticed “flopping,” “diving” and other ways of feigning a foul, Do you think any referees were corrupt, e.g., accepted money to favor one team or another. For the third question about fouls, the midpoint was labelled “just right.”

Similar to the rationale for including questions about the players, these questions were included to control for the possibility that perceptions of FIFA reflect or result from perceptions of the quality of the referees. Referees are the immediate manifestation of the authorities that run a tournament, and participants’ beliefs about FIFA may reflect their perceptions of the quality of the referees. We asked specific questions about offsides and feigning fouls because these can be prominent calls in matches particularly when they involve the negation of a goal (in the case of offsides) or the awarding of a penalty kick (in the case of a foul).

Respondents answered two questions about FIFA: Do you think FIFA does a good job or not organizing the World Cup, deciding about the location, hiring officials, and so forth, and Do you think that the leaders of FIFA are honest or corrupt. For the second FIFA question, the scale points were labelled 1 = generally honest, 3 = neither honest nor corrupt, and 5 = generally corrupt.

### Measures of corruption

The primary focus of the present study was examining relationships between individuals’ perceptions of corruption and societal measures of corruption and the legitimacy of government. We used a measure of corruption from a source other than respondents because we were concerned about the possibility that answering questions about corruption in FIFA might influence perceptions of corruption per se (and the reverse). Moreover, measures of corruption from a source other than the respondents eliminated the possibility that relationships between corruption and other measures could be due to unmeasured individual differences that were related to both corruption and our other measures.

We created a country-level measure of corruption and the legitimacy of government using a series of measures organized by the World Bank [2]. Collectively, these measures are usually referred to as the Worldwide Governance Indicators (WGI). The WGI consists of six indicators: Voice and Accountability, Political Stability and Lack of Violence, Government Effectiveness, Regulatory Quality, Rule of Law, and Control of Corruption. Following inspection of the data that were used to create these indices, the composite measure we created did not include Voice and Accountability because we did not think the content of the measure was sufficiently relevant to corruption to merit inclusion in our study. The definitions of the measures that were included are below. Each describes the state of affairs in a country in 2013. See Treisman [17] for a discussion of measuring societal level corruption.

The World Bank created a standard score for each measure based on a sample of 215 countries. The individual measures represent combinations of various sources. As described by the World Bank: “These aggregate indicators combine the views of a large number of enterprise, citizen and expert survey respondents in industrial and developing countries. They are based on over 30 individual data sources produced by a variety of survey institutes, think tanks, non-governmental organizations, international organizations, and private sector firms.” http://info.worldbank.org/governance/wgi/index.aspx#home.

#### Political stability and absence of violence/terrorism

Reflects perceptions of the likelihood that the government will be destabilized or overthrown by unconstitutional or violent means, including politically-motivated violence and terrorism.

#### Government effectiveness

Reflects perceptions of the quality of public services, the quality of the civil service and the degree of its independence from political pressures, the quality of policy formulation and implementation, and the credibility of the government’s commitment to such policies.

#### Regulatory quality

Reflects perceptions of the ability of the government to formulate and implement sound policies and regulations that permit and promote private sector development.

#### Rule of law

Reflects perceptions of the extent to which agents have confidence in and abide by the rules of society, and in particular the quality of contract enforcement, property rights, the police, and the courts, as well as the likelihood of crime and violence.

#### Control of corruption

Reflects perceptions of the extent to which public power is exercised for private gain, including both petty and grand forms of corruption, as well as "capture" of the state by elites and private interests.

Although each of these measures assessed a different characteristic, we suspected that they were all measures of an underlying entity reflecting stable, reliable, and trustworthy governments and societies. This suspicion was confirmed by a Principal Components Analysis that found that all five measures loaded on a single factor (all loadings above .80). Also, treating all five items as a single scale produced a Cronbach’s alpha of .96. In light of this, we calculated a measure that was the average of these five measures, which we refer to as WB13 (World Bank, 2013) in the analysis below. Note that for this measure higher scores indicate greater political stability, greater government effectiveness, better regulatory quality, stronger rule of law, and more control of corruption.

In terms of using this aggregate instead of the single *Control of Corruption* measure, we should note that corruption has numerous sources and is manifested in many ways [1]. For example, the measure *Rule of Law* includes how well contracts are enforced. Shoddy contract performance is a characteristics of a corrupt society. In corrupt societies, the enforcement of contracts will vary as a function of how the contracted parties are viewed by the civil authorities, which is a prima facie form of corruption. In contrast, in non-corrupt societies contracts are enforced according to the rule of law. The other measures also contain aspects of corruption.

NB: Numerous measures of societal level corruption are available. One of the more popular measures is the Corruption Perceptions Index (CPI) developed and maintained by Transparency International [18]. The present measure WB13 was highly correlated with the CPI for 2013 (at least .95 depending on the exact sample), and as might be expected given this, the results of analyses using the CPI were very similar to the results we report below involving the WB13 measure. Nevertheless, on balance we thought the constructs on which the WB13 was based made it a better measure for our purposes.

### Sample characteristics

A total of 5735 individuals from 81 countries logged onto the website and answered some of the questions. In terms of selecting countries to be included in the analyses, there were countries that could not be included because there were not enough respondents. We included only respondents who resided in countries for which there were 10 or more respondents. This left a total of 5422 respondents.

We realize that 10 is not a particularly stringent criterion for including a country, but we believed that it represented a good compromise among various considerations. Most important, we wanted to include as many countries as possible to maximize the power of our analyses and the representativeness of our sample. Moreover, the analyses we used took into account the number of observations in each country (part of a process known as “Bayes shrinkage”), so that countries with fewer observations influenced parameter estimates less than countries with more observations.

Noting this, although the units of analysis (countries) are weighted, the number of countries still matters, and this is particularly important in terms of the power of analyses that involve level 2 (country-level) measures. So, for hypotheses involving relationships between country-level measures of corruption and country-level means of perceptions of FIFA, the number of level 2 observations (country) is the most important determinant of power. If we had cut at 20 respondents per country, the number of countries in the analyses would have been 23.

We thought that cutting at 20 respondents per country eliminated too many countries in terms of the representativeness of the countries that would have been retained and in terms of statistical power. Including countries that had 10 or more respondents left 29 countries, a number close to 30, an often-cited minimum to provide adequate power to test fixed effects (e.g., Scherbaum [19]). Although more countries would have provided more power, we thought that including countries that had 10 or more respondents was a reasonable balance between including as many countries as possible while reducing the influence of countries that did not have enough respondents to constitute a representative sample (admittedly, with representative defined generously). We should note that the results of analyses that retained countries that had 5 or more respondents (N = 37) were functionally equivalent to the results presented in this paper. The number of respondents for each country for the final sample is presented in Table 1, and the raw country and respondent level data are available as part of the supplemental materials for which the link was provided previously.

**Table 1:** Respondents in final sample by country of residence.

Angola	42
Argentina	30
Austria	19
Belgium	97
Brazil	34
Canada	24
Switzerland	25
Cyprus	45
Germany	1254
Spain	40
France	93
Great Britain	105
Greece	239
Croatia	22
Iran	184
Italy	87
Japan	100
South Korea	155
Luxemburg	10
Mexico	44
Mozambique	42
Netherlands	325
New Zealand	14
Poland	437
Portugal	369
Russian Federation	12
Turkey	379
Ukraine	108
United States	1087

Although the final sample was 5422, the functional sample was closer to 4638 because 784 participants answered only the questions about satisfaction with life and interest in the World Cup. The number of respondents who answered each question varied, and the number of valid responses for each question is included in the descriptive statistics presented in Table 2. Given the focus of this paper on corruption, for questions about the most and least favorite teams, we present statistics only for player corruption and the neutrality of referees.

**Table 2:** Descriptive statistics for survey responses concerning players, officials, and FIFA.

Item	Mean	Variance		Respondents
		Between	Within	
Players…				
Cared about winning	4.24	.07	.49	4604
Skilled	3.66	.09	.59	4584
Problems w/ feigning fouls	3.21	.11	1.03	4559
Officials…				
Overall quality	2.86	.09	.69	4533
Accurate calling offsides	3.37	.05	.73	4489
Too few/too many fouls	2.71	.05	.76	4505
Noticed feigning fouls	2.71	.05	.72	4495
Were any corrupt	2.08	.06	.79	4498
Favorite team…				
Players corrupt	1.36	.04	.36	4098
Officials neutral	2.87	.02	.23	4108
Least favorite team…				
Players corrupt	1.73	.07	.87	2861
Officials neutral	3.36	.07	.43	2881
FIFA…				
Good job organizing	2.58	.06	.97	4549
Officials honest or corrupt	3.72	.16	1.11	4459

Performing a multilevel analysis requires that one set of observations is nested within another. We could have nested individuals in countries in various ways (country of residence, country of citizenship, country of birth), but on balance we thought that country of residence was the most straightforward. Most important, we felt that the norms of one’s country of residence would be the most salient set of norms for individuals. Second, for 81% of respondents, their country of residence was the same as their country of citizenship. Moreover, given that we did not know how long non-citizens had been residing out of their country of citizenship, we could not make some type of adjustment for this. Nevertheless, controlling for whether an individual was a citizen of the country in which he or she resided (represented as a binary measure) did not change any of the results reported below, and we do not report or discuss this aspect of the analyses.

## Results

### Overview of analyses and descriptive statistics

For the primary analyses, we conceptualized the data as a two level structure in which individual respondents were nested within countries of residence, and following the guidelines and procedures described by Nezlek [20], we used the program HLM to conduct a series of multilevel models (MLM). These analyses were conceptually equivalent to conducting a regression analysis for each country and then using the coefficients estimated in these analyses as dependent measures at the next level of analysis.

The first analyses were null models, i.e., no predictors at either level of analysis. These models provided the basic multilevel summary statistics: the estimated mean, the variances at each level of analysis, and the number of valid observations for each measure. These summary statistics also provided a context for evaluating the analyses that focus on the primary hypothesis. Perceptions of FIFA need to be understood within the context of participants’ overall evaluation of the Cup and of their lives in general. For example, did people see the players and officials in a positive light? If FIFA is seen negatively whereas other aspects of the Cup are seen positively this would suggest that evaluations of FIFA are distinct from evaluations of other aspects of the Cup. In turn, this would suggest that evaluations of FIFA do not simply reflect the operation of some global good-bad evaluative mechanism.

These null models are given below. There were *i* people nested within *j* countries. The within-country (level 1) variance is the variance of r_ij_, and the between-country (level 2) variance is the variance of u_0j_.

$$\begin{array}{cc} \text{Within}-\text{country}: & {\text{y}}_{\text{ij}}=\beta_{0\text{j}}+{\text{r}}_{\text{ij}} \end{array}$$

$$\begin{array}{cc} \text{Between}-\text{country}: & \beta_{0\text{j}}=\gamma_{00}+{\text{u}}_{0\text{j}} \end{array}$$

The results of these analyses are summarized in Table 2. As can be seen from the variance estimates in the table, the majority of the variance for each of these measures is at the within-country level, i.e., across individuals. Although means for countries did vary (the column labelled “Between”), individuals within a country varied more from one another than countries varied from one another.

### Overall evaluations of players and match officials

Generally speaking, as can be seen from the means presented in Table 2, the results of these analyses suggested that participants had more positive opinions of the players and the officials than they had of FIFA. In some instances, the distributions of the ratings of the players and the officials were the mirror images of the distributions of the ratings of FIFA. Such differences suggest that participants’ evaluations of FIFA as an organization were distinct from their evaluations of other aspects of the Cup.

In terms of the players, on average, respondents thought that the players were strongly motivated to win and that they were skilled. Respondents also thought that feigning fouls was a problem. Nearly three-quarters of respondents (3359 out of 4559) thought that feigning a foul was a problem (i.e., selected a response of 3 or higher with 3 labeled “a problem”).

Perceptions of the referees were also generally positive. The mean rating of the quality of referees, 2.87, was just below the midpoint of the scale (3), which was labeled acceptable. Although this was significantly less than the midpoint (*p* < .05), the absolute difference was small, and approximately three-quarters of respondents (75.8%) selected option 3, 4, or 5, representing a rating of acceptable or better. In terms of corruption, respondents thought that the referees were generally honest. The mean was 2.08 on a scale with a midpoint of 3 labeled “some were corrupt.” This mean rating was significantly less than the midpoint (*t* = 18.4, *p* < .0001). It is worth noting that of the 4498 people who answered this questions, 1535 (34.1%) selected option 1 labeled “no referees were corrupt,” and 1747 (38.8%) selected option 2. Although these may not be glowing evaluations, taken together, they suggest that respondents were generally satisfied with the quality of the officiating. Less generously, one could conclude that respondents were not dissatisfied.

Although we did not ask a question about corruption among players overall, we did ask questions about corruption of players on respondents’ favorite and least favorite teams. Of the 4098 respondents who answered this question, 3103 (76%) reported that no players were corrupt on their favorite team, and only 5% selected response option 3, 4, or 5 (3 = some were corrupt, 5 = almost all were). As might be expected, respondents’ opinions about the corruption of their least favorite team were not as positive. Of the 2861 respondents who answered this question, just over half (1550, 54%) thought that no players were corrupt on their least favorite team, and 19% selected response option 3, 4, or 5.

We also asked respondents whether they thought the referees were biased toward or against their favorite and least favorite teams. Respondents generally felt their favorites deserve better treatment: they did perceive a slight bias of the officials against the respondent’s own favorite team, a mean of 2.87, which was just below the scale midpoint of 3 (neutral); however, this bias was smaller than the perceived bias of referees in favor of their least favorite team (3.36, *t* = 5.75, *p* < .001).

### Overall perceptions of FIFA

Perceptions of FIFA were not as positive as perceptions of referee and players. In terms of FIFA’s competence in organizing the Cup, respondents felt that FIFA did slightly less than an acceptable job, a mean of 2.60 on a scale with a midpoint of 3 labeled acceptable. Just under half of the 4549 respondents (2036, 44.8%) selected either option 1 or 2 (less than acceptable). Respondents also did not view FIFA positively in terms of corruption. On a scale with a midpoint labeled “neither honest nor corrupt” and an endpoint labeled “generally corrupt” the mean response was 3.73. It is worth noting that out of the 4559 people who answered this question, only 570 (12.6%) selected 1 or 2, the scale points associated with some degree of honesty. 2744 respondents (60.2%) selected 4 or 5, the scale points associated with some degree of corruption–over 30% (1446) selected the maximum 5, which was labeled generally corrupt, and 1242 selected scale point 3, labeled “neither corrupt nor honest”. Although the response scales were different, ratings of corruption for FIFA were almost the mirror image of ratings of corruption for referees.

### Relationships between perceptions of corruption and country level corruption

We examined relationships between perceptions of corruption in football and country level corruption using a series of MLM analyses. The basic model we used is below, and the statistical significance of the relationship between WB13 and the level 1 outcome in the model was estimated by significance of the γ_01_(WB13) coefficient. Keep in mind that higher scores on the WB13 measures indicate more lawfulness, i.e., less corruption. Also, this measure was standardized within the final sample of 29 countries. The results of these analyses are summarized in Table 3.

**Table 3:** Relationships between country level corruption (WB13) and perceptions of FIFA and the World Cup.

	Coefficient	t-ratio	p-level
FIFA corruption	.17	2.38	.024
FIFA organization	-.13	2.93	.007
Interest in World Cup	.12	< 1	ns
Referees’ quality (competence)	.14	2.02	.054
Corruption referees	.00	< 1	ns
Referees’ neutrality favorite team	-.02	< 1	ns
Referees’ neutrality least favorite team	.00	< 1	ns
Corruption favorite team	-.04	1.16	.258
Corruption least favorite team	-.04	< 1	ns

$$\text{Level}\;1:y_{\text{ij}}=\beta_{0\text{j}}+{\text{r}}_{\text{ij}}$$

$$\text{Level}\;2:\beta_{0\text{j}}={\text{γ}}_{00}+{\text{γ}}_{01}(\text{WB}13)+{\text{u}}_{0\text{j}}$$

First, we examined relationships between WB13 and perceptions of the honesty/corruption of FIFA officials, and these analyses found a significant relationship between these two measures. Given the nature of the measures, this coefficient of .17 can be interpreted as follows: For every 1 unit increase in WB13 (and because the *SD* of WB13 was 1.0, this means for every 1 *SD* increase), perceptions of FIFA corruptness went up .17. Keep in mind that the WB13 measure is scored so that higher numbers represent greater rule of law, lower corruption, and so forth. The mean score for the measure of the honesty/corruptness of FIFA officials was 3.73, well above the scale midpoint of 3.0 (*t* = 8.88, *p* < .001). Given that the scale midpoint was labeled “neither honest nor corrupt,” and that scores above the midpoint were associated with varying degrees of corruption, this meant that countries varied in terms of how corrupt (on average) their residents thought FIFA officials were. We should note that no country had a mean score on this variable below 3.0. Following a procedure described by Nezlek [21], the estimated correlation between mean FIFA scores for a country and WB13 was .38.

A similar analysis of how competent FIFA was in organizing the Cup found a negative relationship between WB13 and perceptions of FIFA’s competence. People who lived in countries with greater rule of law (less corruption) saw FIFA as less competent than individuals who lived in countries with weaker rule of law (more corruption). Such a relationship is consistent with the positive relationship between societal corruption and perceptions of corruption in FIFA. Residents of less corrupt countries evaluated FIFA more negatively (more corrupt and less competent) than residents of more corrupt countries. The analysis of the relationship between WB13 and how interested respondents were in the World Cup found a non-significant relationship.

A similar set of analyses of referees’ competence (quality) found a positive relationship between WB13 scores and perceptions of the general competence of referees. People who lived in countries with less corruption saw referees as more competent than individuals who lived in countries with more corruption. Note that this is the reverse relationship than was found between WB13 scores and perceptions of FIFA’s competence. Unlike the results of the analyses of corruption in FIFA, we did not find a significant relationship between WB13 scores and perceptions of how corrupt referees were. There were also no significant relationships between WB13 scores and perceived neutrality of referees toward one’s favorite team or toward one’s least favorite team, and there were no significant relationships between WB13 scores and perceived corruption of players on one’s favorite team or on one’s least favorite team.

### Controlling relationships between country-level corruption and perceptions of corruption in FIFA for individual differences in other measures

We measured a variety of individual differences (background, perceptions of officials and teams) for three reasons. One was to provide a context to evaluate our primary findings. This context was provided by the descriptive statistics presented previously. For example, FIFA was generally viewed as corrupt, whereas referees were not. Second, we collected these additional measures to provide a basis examining, on an exploratory basis, relationships between perceptions of corruption in FIFA and these other measures. Given the lack of quantitative research on perceptions of corruption in FIFA, we thought describing such relationships would be valuable. Third, and most relevant to the primary focus of this article, we wanted to control for the possibility that relationships between perceptions of corruption in FIFA and country-level perception did not reflect these relationships per se but reflected relationships between perceptions of corruption in FIFA and some type of more general negative evaluation (e.g., dissatisfaction with life, perceptions of corruption among referees).

Relationships between perceptions of corruption in FIFA and other individual differences were examined with the following model. The predictor (e.g., satisfaction with life) was entered group mean centered with a random error term. Significance tests were done at level 2 (between country). Was γ_10_, the coefficient representing the mean slope between perceptions of corruption in FIFA and a predictor, significantly different from 0?:

$$\begin{array}{cc} \text{Within}-\text{country}: & {\text{y}}_{\text{ij}}=\beta_{0\text{j}}+\beta_{1\text{j}}(\text{Predictor})+{\text{r}}_{\text{ij}} \end{array}$$

$$\begin{array}{cc} \text{Between}-\text{country}: & \beta_{0\text{j}}=\gamma_{00}+{\text{u}}_{0\text{j}}\\ & \beta_{1\text{j}}=\gamma_{10}+{\text{u}}_{1\text{j}} \end{array}$$

A separate analysis was done for the following predictors: satisfaction with life, interest in the World Cup, perceptions of FIFA’s competence in organizing the World Cup, perceptions of corruption among officials, perceptions of referees’ competence and neutrality, and perceptions of corruption among players on respondents’ favorite and least favorite teams. The results of these analyses are presented in Table 4.

**Table 4:** Individual level relationships between perceptions of corruption in FIFA and other individual difference measures.

	Coefficient	t-ratio	p-level
Satisfaction with life	-0.04	3.23	.003
Interest in World Cup	0.56	2.62	.014
FIFA organization	-0.44	-12.51	.0001
Referees’ quality (competence)	-.23	9.30	.054
Corruption referees	.24	12.87	.0001
Referees’ neutrality favorite team	-.16	3.87	.001
Referees’ neutrality least favorite team	.24	6.70	.0001
Corruption favorite team	.10	2.42	.015
Corruption least favorite team	.04	1.11	ns

As can be seen from the coefficients in Table 4, with the exception of perceptions of corruption among players on the least favorite team, each of these individual differences was significantly related to perceptions of corruption in FIFA. Life satisfaction and perceptions of FIFA’s organization of the World Cup were negatively related to perceptions of corruption in FIFA, whereas interest in the World Cup and perception of corruption among officials and players on the favorite team were positively related to perceptions of corruption in FIFA.

As stated previously, we collected these additional measures to control for the possibility that relationships between perceptions of corruption in FIFA and country level corruption (WB13) reflected relationships between country level corruption and other measures. For most measures, this possibility was precluded by the fact that relationships between these measures and country level corruption were not significant. For the two measures for which this relationship was significant (FIFA organization and referees’ competence), we conducted analyses that controlled ratings of corruption in FIFA for individual differences in these two predictors. This was done by entering these variables as grand-mean centered level 1 predictors (separate analyses for each predictor). Grand-mean centering a level 1 predictor adjusts the intercept for level 2 differences in a predictor [21].

For referees’ competence, the resulting coefficient representing the relationship between the adjusted ratings was .20, which was functionally equivalent to the coefficient from the zero order coefficient between WB13 and perceptions of corruption (.17). When controlling for individual differences in perceptions of FIFA competence, the coefficient between WB13 and perceived corruption in FIFA became non-significant although it remained positive (γ_01_ = .10, *t* = 1.58, p = .12). Similarly, when controlling for individual differences in perceptions of FIFA corruption, the coefficient between WB13 and perceived competence of FIFA became non-significant although it remained negative (γ_01_ = -.05, *t* = 1.27, p = .21).

The estimated correlation between perceptions of FIFA corruption and competence was -.45. This suggests that these measures could be considered to be observed measures of a broader latent construct of “FIFA goodness.” This would not invalidate the observed relationship between WB13 and perceptions of corruption in FIFA; rather, it would suggest that this relationship is part of a broader set of relationships between WB13 and evaluations of FIFA. The fact that perceptions of FIFA organization were not prepotent compared to perceptions of corruption (if anything, changes in the size of coefficients suggests that perceptions of corruption were prepotent), means that perceptions of organization cannot be said to account for relationships between WB13 and perceptions of corruption.

### Additional analyses excluding Germany and the United States

Almost half of our respondents were from Germany and the US, which raises questions about the extent which our results were dominated by relationships in those countries. To address this issue we conducted all the analyses described above on a data set that did not include Germany and the US– 27 countries with about 2500 respondents for most questions. These analyses produced results that were functionally indistinguishable from the results previously reported. For example, in the original analyses that included Germany and the US the mean rating for corruption in FIFA was 3.717, and the coefficient between WB13 and perceptions of corruption in FIFA was .174. When Germany and the US were excluded, the mean rating was 3.709, and the WB13 coefficient was .171 (*t* = 2.23, *p* = .035). Differences between the two samples in the coefficients from other analyses were similarly unimportant.

## Discussion

Countries vary as to rule of law, corruption, effective government, political stability, and governmental regulation. In our sample, these factors were highly correlated, so we combined them into one measure of lawful trustworthiness/corruption. In our sample, citizens living in countries high on lawful trustworthiness (or low on corruption) perceived more corruption in FIFA than citizens living in less lawful and trustworthy societies. These results went against the assimilation hypothesis, which held that living amid corruption would intensify one’s perception of corruption elsewhere. Instead, the findings fit the contrast pattern.

The link between societal corruption and perceiving corruption in FIFA does not mean that people in non-corrupt societies see everyone as more corrupt. There was no relationship between the country’s corruption index (WB13) and perceiving referees or players as corrupt. These non-findings also speak against an assimilation process, which would have led people in corrupt societies to shrug and assume corruption is everywhere. Moreover, overall, participants perceived FIFA as corrupt; they did not see the players or referees as corrupt.

The specificity of perceived corruption (associated with FIFA but not with players or match referees) is consistent with what the media reports had begun to indicate at the time the Cup was held and with what subsequent investigations would establish. Why, then, were the ratings of FIFA’s corruption higher in less corrupt than in more corrupt countries? Establishing the causal process is beyond the scope of our data, but we can speculate. Contrast effects are based on comparison, in this case presumably comparison with everyday life. People in societies that are low on our WB13 measure may be accustomed to assuming widespread fairness and transparency and may regard something like bribery over hosting a football tournament as shockingly outside the norm—whereas people who experience corruption, lawlessness, and abuses of power on a more regular basis may regard such bribery as relatively minor and inconsequential, just part of doing business in everyday life.

People living in countries with low trust, poor rule of law, and ineffective government did not perceive FIFA as incompetent as people living in countries with higher trust, stronger rule of law, and more effective government. The competence findings thus parallel the corruption findings. People in well-run countries rated FIFA as both corrupt and incompetent. People in less well-run countries rated FIFA less negatively on both dimensions. Again, this seems best explained by assuming that people who live with serious problems of government corruption and incompetence may be less shocked by football-related shenanigans, which after all do not have much practical impact on people’s lives, as compared to people who generally expect clean and effective government.

In contrast to the country level relationships between perceptions of FIFA officials and our WB13 measure, we did not find significant country-level relationships between our WB13 measure and ratings of corruption among referees, but perhaps this is not surprising: FIFA officials operate in secret, whereas game referees operate on worldwide television, and so referees’ misdeeds (corruption) might be more difficult to hide. Therefore, it is perhaps reasonable for fans to assume that referees were generally honest and fair. The WB13 ratings were related to the perceived competence of referees (the quality measure), with residents in more corrupt and dysfunctional countries rating referees as less competent than residents in countries with more effective government. That was the closest to any sort of country-level assimilation effect we found.

Although mean ratings of corruption among players indicated that in general, respondents did not think the players were corrupt, there was some acknowledgement that feigning fouls was a problem. Fan bias was readily evident however in that participants gave high marks for honesty to their favorite team but they gave low marks to players on the team they most disliked in terms of corruption. Fan bias was also evident in ratings of referee bias. Consistent with the findings of Hastorf and Cantril [9], fans seemed to think their own team was more virtuous and their opponents less virtuous than what the referee called. They thought the referee was slightly although significantly biased against their favorite team (2.87, where 3.0 was the neutral midpoint representing impartial fairness). They thought the referees were biased in favor of the team they disliked most, and the affirmation of this bias was significantly larger than the bias against their favorite team (3.36 is significantly farther than 2.87 from the 3.0 midpoint).

It also possible that assuming referees are biased in these ways helps fans rationalize the success of teams they dislike: Despised teams advance in the tournament not by playing well but because referees allow them to get away with unfair tactics. This perception was stronger among people with low vs. high life satisfaction. Meanwhile, thinking the referee was biased against one’s favorite team helps explain either why one’s team lost or how particularly good the team needed to be to win despite biased officiating.

### Individual level relationships

As noted by Nezlek [20] relationships at different levels of analysis are mathematically independent, and so it is important to discuss our results at the individual-level of analysis. We found more support for assimilation processes at the individual-level of analysis than at the country-level. We found negative individual-level relationships between life satisfaction and perceptions of corruption in FIFA and among referees. These results suggest that how satisfied people are with their lives influences their perceptions of other entities including institutions. If someone’s life is going well, he or she is more likely to see things in a positive light than if his or her life is not going well. Given the breadth of our measure of life satisfaction we cannot know what specific factors might be responsible for such relationships, but the possibility that life satisfaction is influenced by or influences perceptions of institutions seems plausible and worthy of future study. The positive relationships between perceptions of corruption in FIFA and corruption among the players on one’s favorite team and between perceptions of corruption in FIFA and corruption among referees is also consistent with assimilation processes.

Noting this, such assimilation would need to operate at a different level of analysis than assimilation processes that might exist at a societal level. For example, perceptions of corruption among referees and corruption among players on one’s favorite team were not significantly related to the WB13 measure. When making judgments about referee and player corruption a “local” frame of reference may have been more influential than the more macro frame of reference that may have been more influential when making judgments about FIFA. If so, this would result in greater assimilation. In contrast, relationships between our WB13 measure and perceptions of corruption in FIFA clearly suggested a contrast process.

Our results suggest that assimilation processes existed at the individual level, while at the same time, contrast processes existed at the societal level. To our knowledge, previous research has not examined the possibility that assimilation processes occur at one level of analysis while at the same time contrast processes occur at another. Answering such questions will require research that is designed to do so.

### Limitations and conclusions

Clearly, the generalizability of the present results is limited by the nature of the sample. We did not obtain a stratified random sample in each country, and so we cannot be certain how representative our respondents were of football fans in their respective countries. We are unware of how self-selective motives of respondents might have compromised the validity of out conclusions, but they may have. Similarly, we cannot be certain if our results would have changed if more countries had been included. Despite these shortcomings, we believe that the present results contribute meaningfully to our understanding of perceptions of corruption of international sporting events as the World Cup and the Olympics.

Moreover, perceptions of such events can be important to understanding the in/out group processes that they can engender. Competitors in such international competitions must be citizens of the countries they represent, and national teams can carry the dreams and hopes of their nations. Competitions between national teams can be “good-spirited” and can “bring out the best in us,” or they can be “mean-spirited” and serve as opportunities to revisit ethnic and cultural conflicts. Although FIFA, as an organization, has been quite active in trying to ensure that football matches represent the former and not the latter, such efforts may be undermined by the extent to which FIFA itself is viewed as corrupt. Moreover, recent events regarding the upcoming men’s World Cup in Qatar [22] suggest that corruption among officials remains a problem for FIFA.
